# NCAPG2 Is a Novel Prognostic Biomarker and Promotes Cancer Stem Cell Maintenance in Low-Grade Glioma

**DOI:** 10.3389/fonc.2022.918606

**Published:** 2022-07-08

**Authors:** Wenjun Ren, Shu Yang, Xi Chen, Jishu Guo, Heng Zhao, Ruihan Yang, Zhi Nie, Li Ding, Lei Zhang

**Affiliations:** ^1^ Department of Cardiovascular Surgery, The First People’s Hospital of Yunnan Province, Kunming, China; ^2^ Department of Neurology, The First People’s Hospital of Yunnan Province, Kunming, China; ^3^ Department of Neurosurgery, The Second Affiliated Hospital of Kunming Medical University, Kunming, China; ^4^ Institute for Ecological Research and Pollution Control of Plateau Lakes, School of Ecology and Environmental Science, Yunnan University, Kunming, China; ^5^ Department of Neurology, First Affiliated Hospital of Kunming Medical University, Kunming, China

**Keywords:** low-grade glioma, NCAPG2, prognostic biomarker, immune infiltration, glioma stem cell

## Abstract

Gliomas account for 75% of all primary malignant brain tumors in adults and are associated with high mortality. Mounting evidence has shown that NCAPG2 is differentially expressed in various cancers. However, the prognostic value and immune functions of NCAPG2 in low-grade glioma (LGG) remain unresolved. In the present study, we revealed that NCAPG2 was up-regulated in LGG, and its higher expression was associated with adverse clinical outcomes and poor clinical characteristics, including WHO grade, IDH mutation, 1p/19q codeletion, and primary therapy outcome. The results of the Cox regression analysis revealed that NCAPG2 was an independent factor for the prognosis of low-grade glioma. Meanwhile, we also established a nomogram based on NCAPG2 to predict the 1-, 3-, or 5-year survival in LGG patients. Furthermore, we found that Copy number variation (CNV) and DNA hypomethylation results in its overexpression in LGG. In addition, functional annotation confirmed that NCAPG2 was mainly involved in the immune regulation and WNT signaling pathways. Finally, we determined that increased expression of NCAPG2 was correlated with infiltration levels of various immune cells and immune checkpoint in LGG. Importantly, we found that NCAPG2 was highly expressed in glioma stem cells lines and knockdown of NCAPG2 significantly inhibited the self-renewal ability of GSC. This is the first study to identify NCAPG2 as a new potential prognostic biomarker and characterize the functional roles of NCAPG2 in the progression of LGG, and provides a novel potential diagnostic and therapeutic biomarker for LGG in the future.

## Introduction

Low-grade glioma (LGG) is a relatively common tumor in the central nervous system that mainly includes World Health Organization (WHO) grade 2 and 3 gliomas (1). Mounting evidence has shown that the molecular characteristics of gliomas include mutations of isocitrate dehydrogenase 1 and 2 (IDH1/2) and co-deletion of 1p/19q (2). Due to the various clinical advances in earlier diagnosis and novel therapies, the overall survival has increased, although disparities in access to and outcomes of care for LGG persist. Recently, molecular biomarkers have been shown to be helpful in the diagnosis and prognosis of various cancers. Therefore, uncovering the molecular mechanisms underlying the initiation and progression of LGG and identifying highly reliable biomarkers is crucial to improve the diagnosis and treatment of LGG patients.

NCAPG2 (non-SMC condensin II complex subunit G2), are well characterized for their roles in cell mitosis. It has been shown that NCAPG2 plays an essential role in chromosome condensation and segregation during mitosis (3). Meanwhile, NCAPG2overexpression has also been found to be in hepatocellular carcinoma and its higher expression was associated with adverse clinical outcomes. Forced NCAPG2 expression promotes cell proliferation, migration, and invasion by activating STAT3 and NF-κB signaling pathways in HCC (4). Recently, it was demonstrated that NCAPG2 may be a new therapeutic target and biomarker for future treatment and prognosis in colon cancer (5). However, the expression levels, clinical significance, biological function, and underlying mechanism of NCAPG2 in LGG have not been reported.

In this study, we determined that NCAPG2 was up-regulated in LGG and its higher expression was associated with adverse clinical outcomes and poor clinical characteristics, including WHO grade, IDH mutation, 1p/19q codeletion, and primary therapy outcome. The results of Cox regression analysis revealed that NCAPG2 was an independent factor for LGG prognosis. Meanwhile, we also established a nomogram using NCAPG2 to predict 1-, 3-, or 5-year survival time in LGG patients. Furthermore, the functional annotation confirms that NCAPG2 is mainly involved in the immune response and Wnt signaling pathway. Finally, we uncover that increased expression of NCAPG2 was correlated with infiltration levels of various immune cells and immune checkpoint in LGG. Importantly, we found that NCAPG2 was highly expressed in GBM cell lines. Depletion of NCAPG2 significantly inhibited self-renewal of glioma stem cell (GSC) *in vitro*. In conclusion, this is the first study to characterize the functional roles of NCAPG2 in the progression of LGG, which represents a potential diagnostic and therapeutic biomarker for LGG in the future.

## Materials and Methods

### RNA Sequencing Data and Clinical Data From the TCGA and CGGA Database

We download the RNA expression and clinical data of glioma cohort projects from the The Cancer Genome Atlas (TCGA) research program (https://www.cancer.gov/tcga), including 505 cases of lower-grade glioma (LGG) with clinical information. The various normal tissues expression data obtained from the Genotype-Tissue Expression Project (GTEx) RNA sequencing data as resources (https://www.genome.gov/Funded-Programs-Projects/Genotype-Tissue-Expression-Project). These dataset was utilized analysis the expression of NCAPG2 in glioma and used to examine the correlation between NCAPG2 and various clinical features. We also download the glioma RNA-seq dataset “mRNAseq_693” recruited in the the Chinese Glioma Genome Atlas (CGGA) (http://www.cgga.org.cn/analyse/RNA-data.) contains 693 glioma samples was utilized as verification the prognosis of NCAPG2 in glioma.

### DNA Methylation and CNV Analysis for NCAPG2

Gene Set Cancer Analysis (GSCA) is an integrated platform for genomic, pharmacogenomic, and immunogenomic gene set cancer analysis (6). In this study, we utilized the GSCA to analysis of the correlation between DNA methylation, CNV and NCAPG2 in LGG.

### Function Analysis for NCAPG2 in LGG

In the present research, we utilized the linkedomics database (http://www.linkedomics.org/login.php) obtained the co-expression genes of NCAPG2 in LGG. The gene set kegg.v6.2.symbols.gmt”, which served as a reference gene set, was downloaded from the Molecular Signatures Database (MSigDB) (http://software.broadinstitute.org/gsea/msigdb). We using GSEA software and clusterProfiler package perform the GO and KEGG enrichment analysis signaling pathway of NCAPG2 in LGG (7–9).

### Cox Regression Analysis and Kaplan-Meier Survival Analysis

We utilized cox regression analysis to examine the correlation between NCAPG2 expression and overall survival and disease-specific survival of patients using the TCGA databases. The Kaplan-Meier method was used to assess the difference between high and low risk groups based on the best separation of NCAPG2 expression, employing R packages of survminer and survival.

### Immune Infiltration Analysis

TIMER (https://cistrome.shinyapps.io/timer/) (10), an interactive web portal, could perform comprehensive analysis on the infiltration levels of different immune cells. In this study, we using the TIMER database explored the correlation between NCAPG2 and diverse immune cell infiltration in LGG. We also using GSVA R package to quantify the LGG immune infiltration of 24 tumor-infiltrating immune cells in tumor samples *via* ssGSEA (11).

### Cell Culture Conditions and SiRNA Interference

The NHA cell line was purchased Institute of Medical Biology, Chinese Academy of Medical Sciences. GBM cells lines (including LN229, A172, U251 and Glioma stem cells) were purchased from cell bank of Kunming Institute of Zoology, and cultured in DMEM medium (Corning) supplemented with 10% fetal bovine serum (FBS) and 1% penicillin/streptomycin at 37 °C in atmosphere containing 95% air and 5% CO2.

### Real-Time RT-PCR Assay

The Real-time RT-PCR assay, cells were lysed by RNAiso Plus (Takara Bio, Beijing, China, Cat. 108-95-2). Total RNAs were extracted according to the manufacturer’s protocol, and then reverse transcribed by using RT reagent Kit (Takara Bio, Beijing),. The primer used in this study is as follows: β-actin-F: AAGTGTGACGTGGACATCCGC, β-actin-R: CCGGACTCGTCATACTCCTGCT, NCAPG2-F: TACAAGCCGTGTCTAAGGAGC, NCAPG2-R: TTGAGCCATGTTCGGTTTCCA.

### SiRNA Transfection

NCAPG2 small interfering RNA (siRNA, GenePharma, China) with the corresponding control RNA (siRNA NC) was transfected into cells in logarithmic growth phase. The transfection was performed using the Lipofectamine 3000 transfection reagent (Invitrogen, USA) according to the manufacturer’s protocol. The transfected sequences of NCAPG2 siRNA is: ACTGGAATATCAACTTCAT.

### Tumor Sphere Formation

Cells 3×10^4^/well were plated in ultralow-attachment 6 well plates (Corning; 3471) and grown in serum-free DMEM/F12, supplemented with B27, 20 ng mL^-1^ EGF and 20 ng mL^-1^ bFGF, and 4 g mL^-1^ heparin. The spheres were cultured for 14 days, and then pictured and counted.

### Immunohistochemical Staining (IHC)

For immunohistochemical staining, the sections were deparaffinized in xylene and rehydrated through graded ethanol. Antigen retrieval was performed for 20 min at 95°C with sodium citrate buffer (pH 6.0). After quenching endogenous peroxidase activity with 3% H2O2 and blocking non-specific binding with 1% bovine serum albumin buffer, sections were incubated overnight at 4°C with indicated primary antibodies. Following several washes, the sections were treated with HRP conjugated secondary antibody for 40 min at room temperature, and stained with 3, 3-diaminobenzidine tetrahydrochloride (DAB). Slides were photographed with microscope (Olympus BX43F, Japan). The photographs were analyzed based on the ratio of the staining with the Image-Pro Plus 7.0 software (Media Cybernetics, Inc., Silver Spring, MD, USA). The anti-NCAPG2 used in this manuscript is (ab110882, 1:100).

### Statistical Analysis

Correlation analysis was performed using Pearson correlation test. Kaplan-Meier survival curves were plotted to exhibit the overall survival for LGG patients. Univariate and multivariate Cox regression analyses were used to examine the independent prognostic significance of each variable enrolled in this finding. The significance of the data between two experimental groups was determined by Student’s t-test, and multiple group comparisons were analyzed by one-way ANOVA. P < 0.05 (*), P < 0.01 (**) and P < 0.001 (***), were considered significant.

## Results

### NCAPG2 Was Highly Expressed in LGG

**Figure 1:**
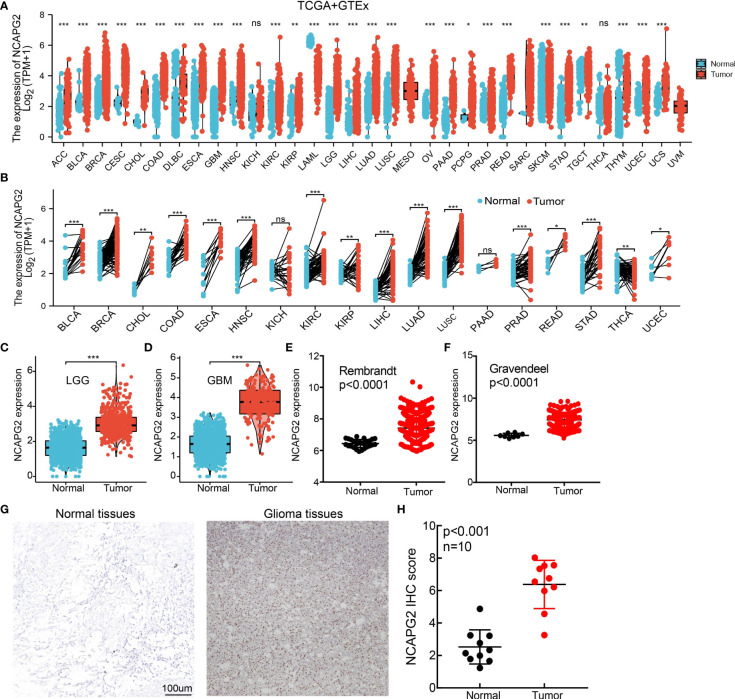
NCAPG2 is highly expressed in LGG. **(A, B)** NCAPG2 expression levels in different tumor tissues and adjacent normal tissues from TCGA and GTEx databases. **(C, D)** The expression of NCAPG2 in LGG and GBM examined by TCGA and GTEx databases. **(E, F)** The expression of NCAPG2 in LGG examine by Rembrandt and Gravendeel dataset. **(G, H)** The protein level of NCAPG2 in Normal and glioma tissues based on IHC assay results. P > 0.05 (NS), P < 0.05 (*), P < 0.01 (**) and P < 0.001 (***).

We explored the mRNA expression levels of NCAPG2 in tumor tissues and adjacent tissues from 33 types of cancer *via* the TCGA dataset and GTEX databases. Our research presented that the mRNA expression levels of NCAPG2 were increased in 28 of the 33 cancers compared with normal tissue (**Figure 1A**). We also examined NCAPG2 expression in paired cancer tissues and adjacent normal tissues in human cancer using TCGA datasets. We found that NCAPG2 expression was significantly higher in 16 of the 18 cancers compared with normal tissue (**Figure 1B**). We used the TCGA and GTEx databases to examine the expression of NCAPG2 in various cancers, the results showed that NCAPG2 increased in LGG, GBM tissue compared to the normal tissues based on TCGA and GTEx datasets (**Figures 1C, D**). Moreove, we utilized the Rembrandt and Gravendeel dataset to validate the expression of NCAPG2 in glioma. Results confirmed that NCAPG2 was upregulated in glioma (**Figures 1E, F**). Finally, the protein level of NCAPG2 was highest in glioma than normal brain tissues based on our IHC assay results (**Figures 1G, H**), consistent with the results from transcriptional analyses.

### Relationship Between NCAPG2 Expression and LGG Clinical Characteristics

**Figure 2:**
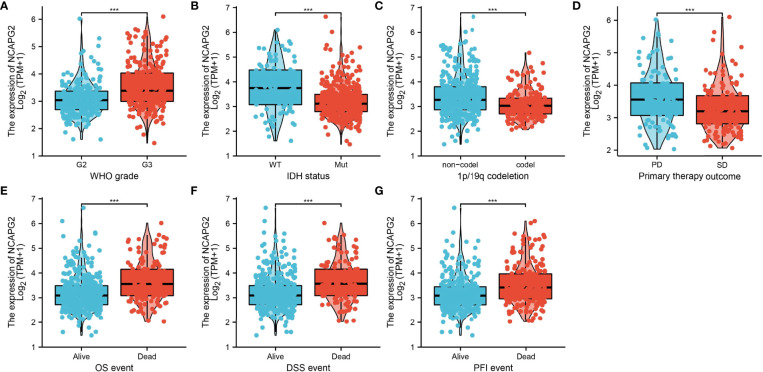
The correlation between NCAPG2 expression and clinical information in LGG. **(A–G)** The correlation between NCAPG2 expression and clinical features, including the higher tumor grades, IDH mutation status, 1p/19q chromosome co-deletion, primary therapy outcome, OS event, DSS event, and PFS event. P < 0.001 (***).

We further explored the correlation between NCAPG2 and clinical characteristics of LGG and we determined that increased NCAPG2 expression was associated with poor clinical characteristics, including higher tumor grade, IDH mutation status, 1p/19q chromosome co-deletion, and primary therapy outcome, OS event, DSS event, and PFS event (**Figures 2A–G**).

In the TCGA-LGG cohort showed increased NCAPG2 expression was significantly correlated with the WHO grade (p<0.001), IDH status (p<0.001), 1p/19q codeletion (p<0.001), OS event (p<0.001), DSS event (p<0.001), PFS event (p<0.001), and age (p<0.001) (**Table 1**).

**Table 1:** Correlation between NCAPG2 and clinic-pathological examined by TCGA-LGG database.

Characteristic	Low expression of NCAPG2	High expression of NCAPG2	p
n	264	264	
WHO grade, n (%)			< 0.001
G2	144 (30.8%)	80 (17.1%)	
G3	88 (18.8%)	155 (33.2%)	
IDH status, n (%)			< 0.001
WT	29 (5.5%)	68 (13%)	
Mut	233 (44.4%)	195 (37.1%)	
1p/19q codeletion, n (%)			< 0.001
codel	109 (20.6%)	62 (11.7%)	
non-codel	155 (29.4%)	202 (38.3%)	
Primary therapy outcome, n (%)			< 0.001
PD	34 (7.4%)	76 (16.6%)	
SD	69 (15.1%)	77 (16.8%)	
PR	40 (8.7%)	24 (5.2%)	
CR	84 (18.3%)	54 (11.8%)	
Race, n (%)			0.560
Asian	3 (0.6%)	5 (1%)	
Black or African American	9 (1.7%)	13 (2.5%)	
White	245 (47.4%)	242 (46.8%)	
Age, n (%)			0.068
<=40	143 (27.1%)	121 (22.9%)	
>40	121 (22.9%)	143 (27.1%)	
Histological type, n (%)			0.084
Astrocytoma	86 (16.3%)	109 (20.6%)	
Oligoastrocytoma	68 (12.9%)	66 (12.5%)	
Oligodendroglioma	110 (20.8%)	89 (16.9%)	
OS event, n (%)			< 0.001
Alive	222 (42%)	170 (32.2%)	
Dead	42 (8%)	94 (17.8%)	
DSS event, n (%)			< 0.001
Alive	224 (43.1%)	173 (33.3%)	
Dead	37 (7.1%)	86 (16.5%)	
PFI event, n (%)			< 0.001
Alive	186 (35.2%)	132 (25%)	
Dead	78 (14.8%)	132 (25%)	
Age, meidan (IQR)	39 (31, 50.25)	42.5 (33, 54.25)	0.017

G2, grade II; G3, grade III; PD, progressive disease; SD, stable disease; PR, partial response; CR, complete response; OS event, Overall survival event; DSS event, disease specific survival; and PFI event, progression –free survival.

**Figure 3:**
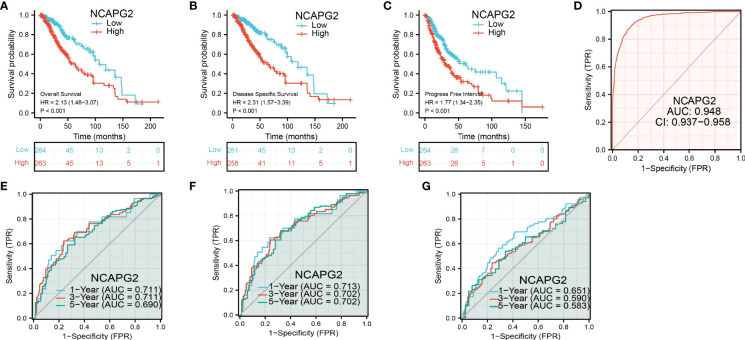
Prognostic and diagnostic value of NCAPG2 **(A-C)** Kaplan–Meier survival curves showed that LGG patients with high NCAPG2 expression exhibited poor overall survival, disease-specific survival and progression-free survival of NCAPG2 in LGG determined by TCGA-LUAD dataset. **(D-G)** Time-dependent ROC curves were used to determine the diagnostic value of NCAPG2 in LGG.

Based on the univariate analysis by logistic regression, we determined that increased NCAPG2 expression was significantly correlated with WHO grade (G3 vs. G2), 1p/19q codeletion (non-codel vs. codel), primary therapy outcome (PR&CR vs. PD&SD), IDH mutation status (Mut vs. WT), and histological type (oligodendroglioma vs. oligoastrocytoma) (**Table 2**). Given that NCAPG2 was increased in LGG and its higher expression was associated with poor clinical characteristics. Therefore, we further explored the prognostic value of NCAPG2 in LGG. Patients with LGG patients were divided into high- or low-expression groups based on the median expression value. Increased expression of NCAPG2 was associated with adverse clinical outcomes, including poor overall survival, disease-specific survival, and progression-free survival (**Figures 3A–C**). Receiver operator characteristic (ROC) curve analysis showed that AUC values of NCAPG2 was 0.948 in the TCGA-LGG dataset (**Figure 3D**).

**Table 2:** Correlation between NCAPG2 and clinic-pathological characteristics examined by logistic regression.

Characteristics	Total(N)	Odds Ratio(OR)	P value
WHO grade (G3 vs. G2)	467	3.170 (2.178-4.645)	<0.001
1p/19q codeletion (non-codel vs. codel)	528	2.291 (1.579-3.348)	<0.001
IDH status (Mut vs. WT)	525	0.357 (0.219-0.568)	<0.001
Primary therapy outcome (SD vs. PD)	256	0.499 (0.295-0.834)	0.009
Histological type (Oligodendroglioma vs. Astrocytoma)	394	0.638 (0.428-0.949)	0.027

**Figure 4:**
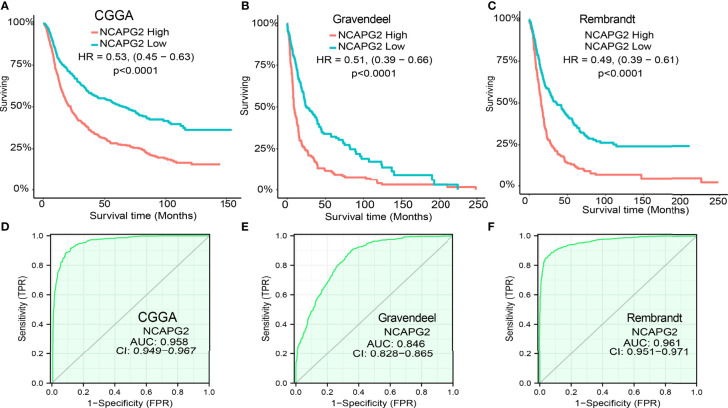
Validation of the prognostic and prognostic value of NCAPG2 in glioma. **(A–C)** Validation of the prognosis of NCAPG2 in glioma by CGGA, Rembrandt and Gravendeel datasets **(D–F)** ROC analyses revealed the predictive value of NCAPG2 in glioma based on CGGA, Rembrandt and Gravendeel datasets.

According to time-dependent ROC, the NCAPG2 expression level had a relatively good performance in predicting 1-year (C statistics, 0.711), 3-year (C statistics, 0.711), and 5-year overall survival (C statistics, 0.690) in LGG patients (**Figure 3E**), had a better performance in predicting 1-year (C statistics, 0.713), 3-year (C statistics, 0.702), and 5-year disease-free survival (C statistics, 0.702) in LGG patients (**Figure 3F**), and had a relatively good performance in predicting 1-year (C statistics, 0.651), 3-year (C statistics, 0.590), and 5-year progression-free survival (C statistics, 0.583) in LGG patients (**Figure 3G**). To validate the prognosis of NCAPG2 in LGG, we analyzed of CGGA, Gravendeel, and Rembrandt dataset and found that upregulation of NCAPG2 was correlated with poor prognosis in patients with LGG (**Figures 4A–C**). Receiver operator characteristic (ROC) curve analysis showed that receiver operating characteristics curve (AUC) values of NCAPG2 were 0.958, 0.846, and 0.961 in the CGGA, Gravendeel, and Rembrandt dataset (**Figures 4D–F**).

### Univariate and Multivariate Cox Regression Analyses of Different Parameters on Overall Survival

We also performed a univariate analysis of the prognostic factors for overall survival. We found that high expression of NCAPG2 was associated with higher tumor grades, IDH mutation status, 1p/19q chromosome co-deletion, and age (**Table 3**). Furthermore, we used the Cox regression model for multivariate analysis. Results demonstrated that NCAPG2 expression, tumor grades, IDH mutation status, and 1p/19q chromosome co-deletion, and age were independent risk factors for overall survival **(** **Table 3** **)**.

**Table 3:** Examine the prognosis of NCAPG2 in LGG patients analysis by cox regression.

Characteristics	Total(N)	Univariate analysis		Multivariate analysis	
		Hazard ratio (95% CI)	P value	Hazard ratio (95% CI)	P value
WHO grade	466				
G2	223				
G3	243	3.059 (2.046-4.573)	<0.001	1.991 (1.280-3.097)	0.002
IDH status	524				
WT	97				
Mut	427	0.186 (0.130-0.265)	<0.001	0.341 (0.214-0.543)	<0.001
1p/19q codeletion	527				
codel	170				
non-codel	357	2.493 (1.590-3.910)	<0.001	1.703 (1.008-2.878)	0.047
Histological type	332				
Oligoastrocytoma	134				
Oligodendroglioma	198	0.845 (0.527-1.352)	0.482		
Age	527				
<=40	264				
>40	263	2.889 (2.009-4.155)	<0.001	2.872 (1.875-4.400)	<0.001
Gender	527				
Female	238				
Male	289	1.124 (0.800-1.580)	0.499		
NCAPG2	527	1.763 (1.465-2.121)	<0.001	1.302 (1.032-1.642)	0.026

### Predictive Value of the NCAPG2 Level Based on Clinical Subgroups

**Figure 5:**
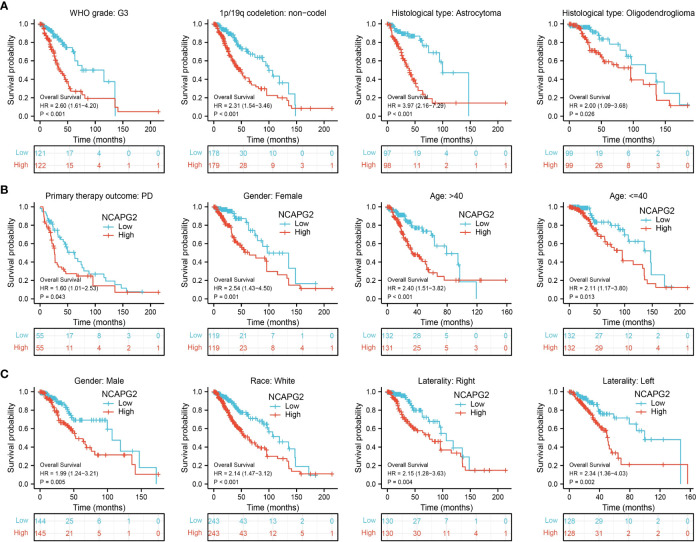
Associations between NCAPG2 expression level and the overall survival in different clinical subgroups of LGG in the TCGA database. **(A–C)** including the WHO grade 3, 1p/19q non-codeletion, astrocytoma, Oligodendroglioma, subgroup of PD, Male, Female, age >40, age <40, Race, white, Lateralty, right and left.

To validate the robustness of our findings, we subsequently investigated the correlations between NCAPG2 expression and OS across different subgroups stratifying patients by various clinical features. The results consistently showed that glioma patients with a higher NCAPG2 expression had a significantly deteriorative OS compared to those with a low NCAPG2 level, including the subgroup of WHO grade 3, 1p/19q non-codeletion, astrocytoma, Oligodendroglioma, subgroup of PD, Male, Female, age >40, age <40, Race, white, Lateralty, right and left (**Figures 5A–C**).

### Prognostic Model Based on NCAPG2 Expression in LGG

**Figure 6:**
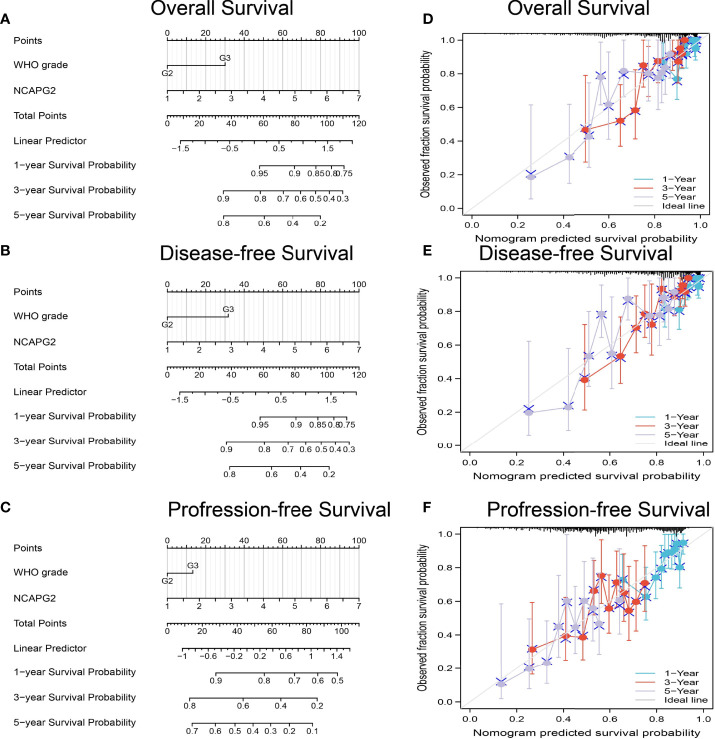
Construction a nomogram to predicted the prognosis of NCAPG2 in LGG. Construction a nomogram to predicted the **(A)** OS, **(B)** DSS, and **(C)** PFI in LGG patients. The calibration curve used to display the TCGA-LGG cohort for **(D)** OS, **(E)** DSS and **(F)** PFI.

We used the NCAPG2 expression and tumor grades to construct a prognostic nomogram, and a calibration curve was drawn to test the efficiency of the nomogram. This nomogram was then used to predict overall survival, disease-specific survival, and the progression-free surviv (**Figures 6A–C**). The calibration curves also indicated the desirable prediction of the three nomograms for the 1-, 3-, and 5-year clinical outcomes (**Figures 6D–F**). These results confirmed that this prognostic model could accurately predict the prognosis of LGG patients.

### CNV and DNA Methylation Analysis of NCAPG2 in LGG

**Figure 7:**
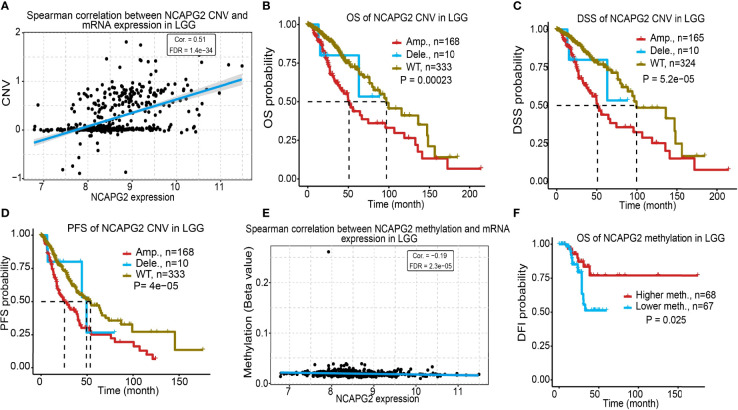
Analysis of correlation between CNV, DNA methylation and NCAPG2 expression in LGG. **(A)** Analysis of correlation between CNV and NCAPG2 expression in LGG. **(B–D)** CNV of NCAPG2 was significantly correlated to OS, DSS and PFS in LGG. **(E)** Analysis of correlation between DNA methylation and NCAPG2 expression in LGG. **(F)** DNA methylation of NCAPG2 was significantly correlated to OS in LGG.

CNA is a common genetic alteration associated with the occurrence and progression of cancer by modulating the expression of tumor-related genes (12). To explore the mechanisms underlying NCAPG2 dysregulation in LGG, we retrieved online sequencing data from the TCGA database. We examine the correlation between copy number variation (CNV) and NCAPG2 in LGG. We found that a positive correlation between CNV and NCAPG2 mRNA expression in LGG (**Figure 7A**). Next, we assessed the prognostic value of NCAPG2CNV in terms of LGG and overall survival. Results confirmed that the NCAPG2 copy-number-altered group was associated with poorer OS, DSS and PFS in LGG compared to the unaltered group (**Figures 7B–D**).

DNA methylation is crucial for the epigenetic regulation of gene expression. To elucidate the mechanisms of abnormal overexpression of NCAPG2 in LGG tissues, we examined the correlation between DNA methylation and NCAPG2 expression in LGG using different public databases. First, we found that the methylation level was negatively correlated with the expression of NCAPG2 in LGG (**Figure 7E**). Importantly, using the methsurv statistical tool, we found that the decreased level of methylation was correlates with worse prognosis in the TCGA-LGG dataset (**Figure 7F**).

### Functional Analysis of NCAPG2 in LGG

**Figure 8:**
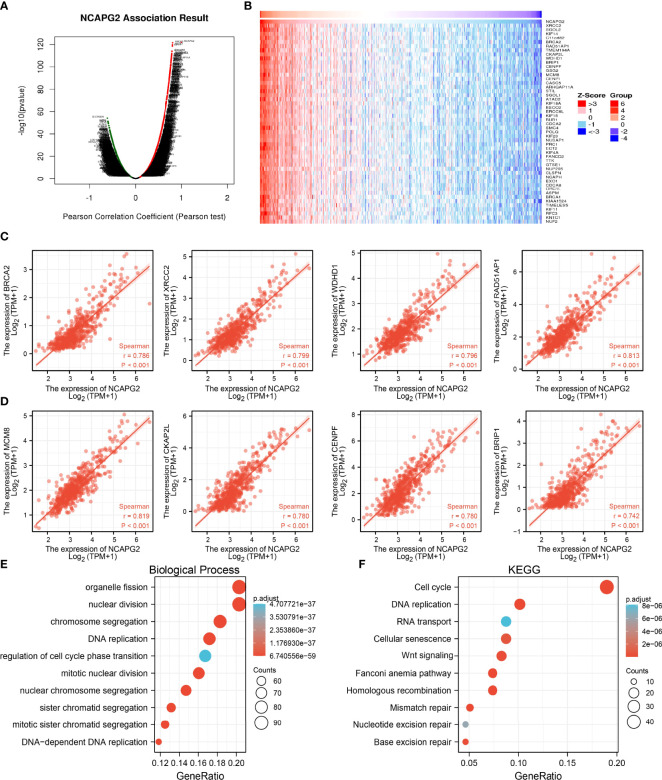
Analysis the function of NCAPG2 expression in LGG. **(A–D)** Analysis the co-expression genes of NCAPG2 in LGG examined by linkomics databases. **(E)** Analysis the biology process involved by NCAPG2 in LGG. **(F)** Analysis the KEGG signaling pathway of NCAPG2 in LGG.

To explore the potential functions of NCAPG2 in the progression of LGG, using linkedomics tools, we obtained co-expressed genes that were positively correlated with that of NCAPG2 in LGG (**Figures 8A–D**). Furthermore, functional annotation showed that NCAPG2 was involved primarily in organelle fission, nuclear division, chromosome segregation, DNA replication, regulation of cell cycle phase transition, and mitotic nuclear division among the Gene Ontology (GO) annotation terms (**Figure 8E**). NCAPG2 participates mainly in the Cell cycle, DNA replication, RNA transport, Cellular senescence, and Wnt signaling pathway (**Figure 8F**).

### NCAPG2-Related Signaling Pathways Identified Using Gene Set Enrichment Analysis

**Figure 9:**
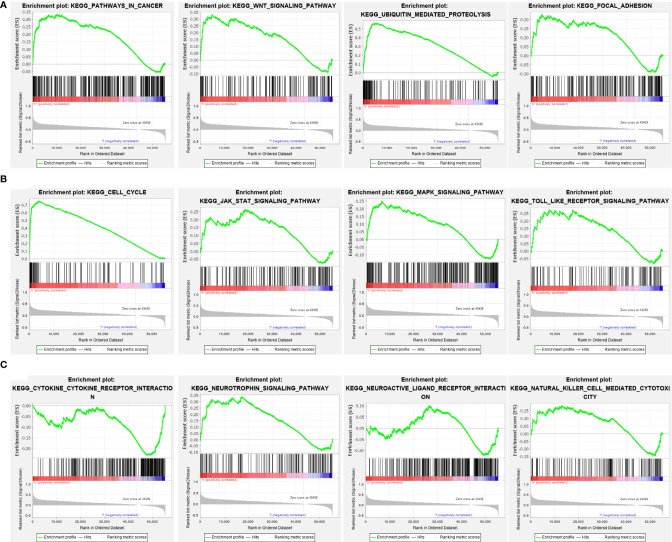
KEGG signaling pathway explore by GSEA software. **(A–C)** The signaling pathway involved by NCAPG2 in LGG examined by GSEA software.

To further explore the molecular mechanisms of NCAPG2 involvement in LGG, we performed a gene set enrichment analysis (GSEA). High expression of NCAPG2 was mainly associated with the pathway in cancer, WNT signaling pathway, ubiquitin mediated proteolysis, focal adhesion, cell cycle, JAK/STAT signaling pathway, MAPK signaling pathway, Toll-like receptor signaling pathway, cytokine receptor interactions, neuroactive ligand receptor interactions, and natural killer cells mediated cytotoxicity (**Figures 9A–C**).

### Association Between NCAPG2 Expression Levels and Immune Cell Infiltration in LGG

**Figure 10:**
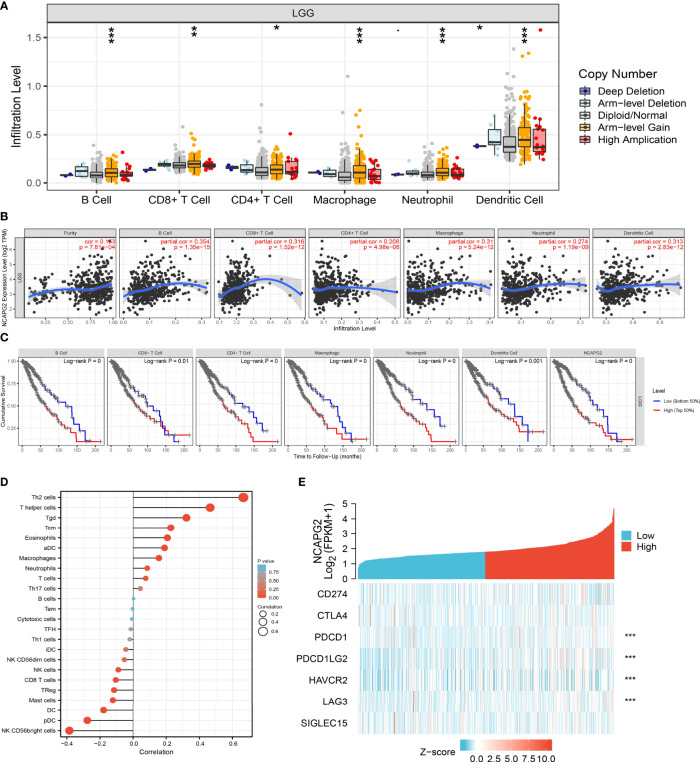
Analysis of the correlation between NCAPG2 expression and diverse immune cell infiltration. **(A)** The correlation between NCAPG2 expression and somatic copy number alterations examine by TIMER. **(B)** The correlation between NCAPG2 expression and the infiltration levels of B cells, CD4+ T cells, CD8+ T cells, dendritic cells, Macrophages and Neutrophils. **(C)** The B cells, CD4+ T cells, CD8+ T cells, dendritic cells, Macrophages and Neutrophils are correlated with the cumulative survival rate in LGG examine by TIMER. **(D)** The correlation between NCAPG2 expression and 24 type immune cell infiltration. **(E)** The correlation between the NCAPG2 expression and various immune checkpoints related genes. P < 0.05 (*), P < 0.01 (**) and P < 0.001 (***).

Given that GSEA enrichment analysis above showed that NCAPG2 may be correlated with immune response regulation, we therefore examined the association between NCAPG2 expression levels and immune cell infiltration, we found that different alterations in NCAPG2 somatic copy number significantly affected the infiltration levels of various immune cells, including B cells, CD4+ and CD8+ T cells, neutrophils, and dendritic cells (**Figure 10A**). Furthermore, we used the TIMER database to examine the correlation between NCAPG2 expression and immune infiltration levels for various immune cells in LGG. The results indicated that NCAPG2 was significantly correlated with the level of B cells, CD8+ T cells, CD4+ T cells, macrophages, neutrophils, and dendritic cells, and negatively correlated with tumor purity (**Figure 10B**). Our analysis using the Cox proportional hazard model demonstrated that B cells, CD8+ and CD4+ T cells, macrophages, neutrophils, dendritic cells, and the expression of NCAPG2 were significantly associated with worse overall survival in LGG patients (**Figure 10C**).

Furthermore, we used ssGSEA to quantify the level of immune cell infiltration in the high- and low-expression groups of NCAPG2. We determined that increased expression of NCAPG2 was positively and negatively associated with the abundance of 24 immune cells (**Figure 10D**). Given that immune checkpoints play a crucial role in tumor immunosuppression, we analyzed the correlation between NCAPG2 expression and that of the immune checkpoint-related genes CD274, CTLA4, HAVCR2, LAG3, PDCD1, PDCD1LG2, TIGIT, and SIGLEC15 in LGG using Pearson’s correlation analysis. NCAPG2 expression was significantly positively correlated with the expression of PDCD1, PDCD1LG2, HAVCR2, and LAG3 in this analysis (**Figure 10E**). These results confirmed that NCAPG2 played a crucial role in immune infiltration in LGG.

### NCAPG2 Is Crucial for GSC Maintenance

**Figure 11:**
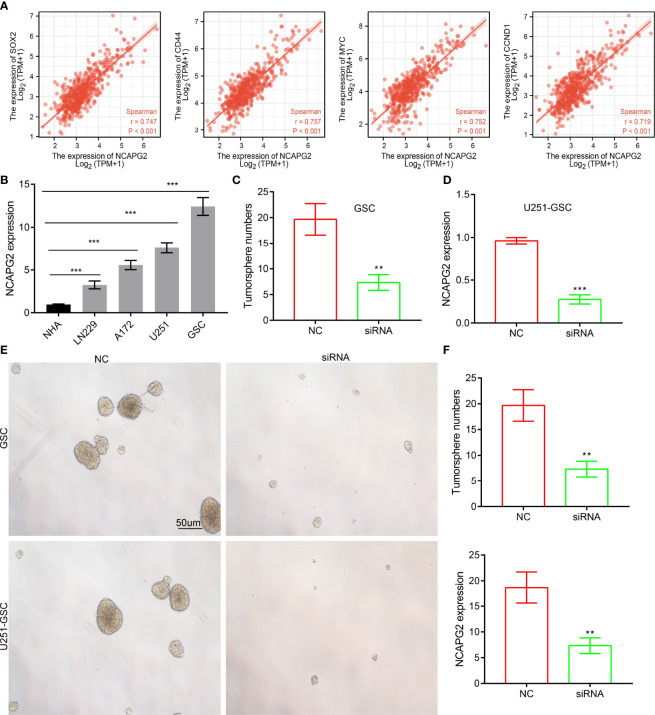
Depletion of NCAPG2 inhibits the self-renewal ability of GSC. **(A)** Correlation between NCAPG2 expression and glioma stem cell marker genes, including Sox2, CD44, MYC and CCND1. **(B)** The expression of NCAPG2 in normal human astrocytes cells (NHA) and GBM cell lines. **(C, D)** The qRT-PCR assay showed that the expression of NCAPG2 mRNA was significantly decreased in U251-GSC and GSC cells after treatment with targeted siRNA. **(E, F)** NCAPG2 knockdown significantly inhibited GSC the self-renewal ability. P < 0.01 (**) and P < 0.001 (***).

Given that GSEA enrichment analysis above showed that NCAPG2 may be correlated with WNT signaling pathway, we therefore examined whether NCAPG2 affect self-renewal of glioma stem cell (GSC) *in vitro*. we performed the co-expression analysis and confirmed that CAPG2 positively correlates with the expressions of well-defined glioma stem cell marker genes, including Sox2, CD44, MYC and CCND1 (**Figure 11A**). Furthermore, we detected NCAPG2 expression levels in GBM cells using a qRT-PCR assay. Results confirmed that NCAPG2 was significantly increased in GBM cell lines, especially in GSC cells (**Figure 11B**). The qRT-PCR assay showed that the expression of NCAPG2 mRNA was significantly decreased in U251-GSC and GSC cells after treatment with targeted siRNA (**Figures 11C, D**). Finally, we then used GSC and U251-GSC to validate the potential role of NCAPG2 in GSC maintenance. We found that knockdown of NCAPG2 was significantly inhibited the self-renewal ability of GSC (**Figures 11E, F**).

## Discussion

Grades I and II are grouped as LGGs, while grades III and IV as high-grade gliomas, LGGs have a 10- to 15-year survival. Although earlier diagnosis and newer therapies have increased overall survival, disparities in access to and outcomes of care for LGGs persist (13). Therefore, more sensitive and specific diagnostic biomarkers and potential therapeutic targets for this type of cancer need to be identified.

NCAPG2, as a member of the Sorting nexins proteins family, plays an indispensable role in protein sorting and transport (14). In this study, we determined that NCAPG2 was up-regulated in various human cancers and especially in LGG. Meanwhile, increased expression of NCAPG2 was associated with poor clinical characteristics, including higher tumor grades, histological type, IDH mutation status, 1p/19q chromosome co-deletion, and primary therapy outcome. More importantly, we found that higher expression of NCAPG2 was correlated with poor clinical outcome, including overall survival, disease-specific survival, and progression-free survival. We also performed a univariate analysis including the prognostic factors for overall survival. We found that high expression of NCAPG2 was associated with higher tumor grades, histological type, IDH mutation status, 1p/19q chromosome co-deletion, and primary therapy outcome. Furthermore, we utilized the Cox regression model for multivariate analysis; the results demonstrated that NCAPG2 expression, tumor grades, IDH mutation status, and primary therapy outcome may be an independent risk factor for overall survival. TCGA-LGG cohort also confirmed that increased NCAPG2 expression was significantly correlated with the WHO grade (p<0.001), IDH status (p<0.001), 1p/19q codeletion (p<0.001), OS event (p<0.001), DSS event (p<0.001), PFS event (p<0.001), and age (p<0.001) (**Table 1**).

A growing number of studies have reported that CNV and DNA methylation plays an important role in gene expression regulation (15, 16). In this study, we found that a positive correlation between CNV and NCAPG2 mRNA expression in LGG. Next, we assessed the prognostic value of NCAPG2CNV in terms of LGG and overall survival. Results confirmed that the NCAPG2 copy-number-altered group was associated with poorer OS, DSS and PFS in LGG compared to the unaltered group. DNA methylation is crucial for the epigenetic regulation of gene expression. We found that the methylation level was negatively correlated with the expression of NCAPG2 in LGG. Importantly, using the methsurv statistical tool, we found that the decreased level of methylation was correlates with worse prognosis in the TCGA-LGG dataset. Although various molecular mechanisms can lead to increased gene expression, such as the lncRNA/miRNA axis, regulation of transcription factors and gene copy number amplification, CNV and DNA hypomethylation is one of the main regulatory mechanisms of gene expression.

Previous studies reported that NCAPG2 is necessary for the cell mitosis (17). In our study, GO enrichment analyses indicated that NCAPG2 participated primarily in biological processes, including organelle fission, nuclear division, chromosome segregation, DNA replication, regulation of cell cycle phase transition, and mitotic nuclear division. GSEA enrichment analysis confirmed that NCAPG2 is primarily involved in cancer, WNT signaling pathway, ubiquitin mediated proteolysis, focal adhesion, cell cycle, JAK/STAT signaling pathway, MAPK signaling pathway, Toll-like receptor signaling pathway, cytokine receptor interactions, neuroactive ligand receptor interactions, and natural killer cells mediated cytotoxicity

Previous reports have suggested that NCAPG2 expression is strongly correlated with immune infiltration levels in lung adenocarcinoma progression (18, 19). CD8 + tumor infiltrating lymphocytes have also been shown to be associated with glioma prognosis (20). In this research, we revealed that NCAPG2 somatic copy number alterations affected infiltration levels of various immune cells, including B cells, CD4+ and CD8+ T cells, neutrophils, and dendritic cells. Furthermore, we found that NCAPG2 was significantly correlated with the level of B cells, CD8+ T cells, CD4+ T cells, macrophages, neutrophils and dendritic cells, and was negatively correlated with tumor purity. More importantly, our results confirmed that NCAPG2 expression was significantly positively correlated with the expression of HAVCR2, LAG3, PDCD1, and PDCD1LG2in LGG. Given the effects of NCAPG2 on LGG immune cell infiltration, we can infer that increased expression of NCAPG2 may promote mast cell infiltration and contribute to a poor prognosis. Therefore, our results demonstrated that NCAPG2 might affect immune cell infiltration, making them a predictive biomarker for immunotherapy in LGG patients.

Overexpression of NCAPG2 promotes cell proliferation and migration abilities of NSCLC cells (19). However, no studies have reported the functions of NCAPG2 in LGG. In this study, we revealed that NCAPG2 was highly expressed in GBM cell lines, especially in GSC cells. Depletion of NCAPG2 significantly inhibited GSC the self-renewal ability. In summary, this was the first study to characterize the functional roles of NCAPG2 in the progression of LGGs, which provides potential diagnostic and therapeutic biomarkers for LGG in the future. Collectively, these results highlight that NCAPG2 plays a dual role in tumorigenesis and progression.

To the best of our knowledge, this is the first study to explore the correlation between NCAPG2 and LGG. However, there are some limitations to our research. First, our study was based on expression data extracted from TCGA but maybe more convincing if supported by a prospective clinical study. Furthermore, the biological functions of NCAPG2 need to be further explored *in vivo* experiments. In the future, we will pay more attention to the function of NCAPG2 in tumor progression and tumor microenvironment regulation of LGG. Furthermore, we will perform more *in vivo* and vitro experiments to explore the function and the potential molecular mechanisms of NCAPG2 in tumor progression and tumor microenvironment regulation of LGG.

## Conclusions

Our findings confirmed that DNA hypomethylation-induced increased expression of NCAPG2 in LGG. Furthermore, increased expression of NCAPG2 was positively correlated with immune cell infiltration and immune checkpoints. Finally, depletion of NCAPG2 significantly inhibited GSC the self-renewal ability. This is the first study to identify NCAPG2 as a new potential prognostic biomarker and to characterize the functional roles of NCAPG2 in the progression of LGG, providing potential diagnostic and therapeutic biomarkers for LGG in the future.

## Data Availability Statement

The original contributions presented in the study are included in the article/supplementary material. Further inquiries can be directed to the corresponding authors.

## Author Contributions

XC, SY, and JG designed this work and performed related assay. HZ, RY, and ZN analyzed the data. LD and LZ supervised and wrote the manuscript. All authors contributed to the article and approved the submitted version.

## Funding

This work was supported by China International Medical Foundation: Cerebrovascular Disease Youth Innovation Fund (Grant No.Z-2016-20-2101)

## Conflict of Interest

The authors declare that the research was conducted in the absence of any commercial or financial relationships that could be construed as a potential conflict of interest.

## Publisher’s Note

All claims expressed in this article are solely those of the authors and do not necessarily represent those of their affiliated organizations, or those of the publisher, the editors and the reviewers. Any product that may be evaluated in this article, or claim that may be made by its manufacturer, is not guaranteed or endorsed by the publisher.
